# Predicting Publication of Clinical Trials Using Structured and Unstructured Data: Model Development and Validation Study

**DOI:** 10.2196/38859

**Published:** 2022-12-23

**Authors:** Siyang Wang, Simon Šuster, Timothy Baldwin, Karin Verspoor

**Affiliations:** 1 School of Computing and Information Systems University of Melbourne Melbourne Australia; 2 Mohamed bin Zayed University of Artificial Intelligence Abu Dhabi United Arab Emirates; 3 School of Computing Technologies RMIT University Melbourne Australia

**Keywords:** clinical trials, study characteristics, machine learning, natural language processing, pretrained language models, publication success

## Abstract

**Background:**

Publication of registered clinical trials is a critical step in the timely dissemination of trial findings. However, a significant proportion of completed clinical trials are never published, motivating the need to analyze the factors behind success or failure to publish. This could inform study design, help regulatory decision-making, and improve resource allocation. It could also enhance our understanding of bias in the publication of trials and publication trends based on the research direction or strength of the findings. Although the publication of clinical trials has been addressed in several descriptive studies at an aggregate level, there is a lack of research on the predictive analysis of a trial’s publishability given an individual (planned) clinical trial description.

**Objective:**

We aimed to conduct a study that combined structured and unstructured features relevant to publication status in a single predictive approach. Established natural language processing techniques as well as recent pretrained language models enabled us to incorporate information from the textual descriptions of clinical trials into a machine learning approach. We were particularly interested in whether and which textual features could improve the classification accuracy for publication outcomes.

**Methods:**

In this study, we used metadata from ClinicalTrials.gov (a registry of clinical trials) and MEDLINE (a database of academic journal articles) to build a data set of clinical trials (N=76,950) that contained the description of a registered trial and its publication outcome (27,702/76,950, 36% published and 49,248/76,950, 64% unpublished). This is the largest data set of its kind, which we released as part of this work. The publication outcome in the data set was identified from MEDLINE based on clinical trial identifiers. We carried out a descriptive analysis and predicted the publication outcome using 2 approaches: a neural network with a large domain-specific language model and a random forest classifier using a weighted bag-of-words representation of text.

**Results:**

First, our analysis of the newly created data set corroborates several findings from the existing literature regarding attributes associated with a higher publication rate. Second, a crucial observation from our predictive modeling was that the addition of textual features (eg, eligibility criteria) offers consistent improvements over using only structured data (*F*_1_-score=0.62-0.64 vs *F*_1_-score=0.61 without textual features). Both pretrained language models and more basic word-based representations provide high-utility text representations, with no significant empirical difference between the two.

**Conclusions:**

Different factors affect the publication of a registered clinical trial. Our approach to predictive modeling combines heterogeneous features, both structured and unstructured. We show that methods from natural language processing can provide effective textual features to enable more accurate prediction of publication success, which has not been explored for this task previously.

## Introduction

### Background

Rigorously conducted randomized controlled trials provide the highest level of scientific evidence, enabling medical practitioners to provide better care for patients and ultimately improving public health. Available, findable, and accessible clinical research results are necessary for the successful transfer of findings into evidence-based practice and further research [1]. In recent years, improved clinical trial registration has meant that more trials than ever are now discoverable and searchable according to a variety of metadata. However, registration does not offer detailed information about important aspects of the study execution and results, such as specification of outcomes and pointers to all resulting publications [2]. Scientific publications resulting from completed clinical trials offer a means of disseminating the findings comprehensively, which is essential for supporting subsequent clinical trials, increasing possibilities for research collaboration, and advancing medical practice and research [3]. In addition to research results, detailed information about the study methods provided in publications is also critical to appraising the validity, reliability, and applicability of clinical evidence in clinical practice [4].

Despite the importance of publication, many clinical trials are never published. Estimates of the publication rate of trials vary depending on the medical area and length of the follow-up period. Broadly, publication rates are in the range of 52% to 77% [5-8]. On the basis of a shorter follow-up period of 30 months from clinical trial completion, the rates tend to be lower, at approximately 11% to 46% [3,6,9]. When results are not published, are substantially delayed, or are published selectively based on the direction or strength of the findings, the ability of health care professionals and consumers to make informed decisions based on the full body of current evidence is impeded [10,11]. Such gaps in the evidence base can lead to the use of ineffective or harmful interventions and potentially waste scarce health care resources. In a study by Eyding et al [12] on the treatment of depression, it was found that, when unpublished studies were included in a meta-analysis, the antidepressant reboxetine had more adverse effects but no better efficacy than placebo for treatment of major depression, a different finding from that when only published studies were included. Additional ethical concerns have also been raised by some researchers [7,13], highlighting that, in the case of nonpublication, the trial participants are still exposed to the risks of participation but without the societal benefits resulting from the dissemination of study results.

In this work, we explore the factors affecting publication of the outcomes of individual clinical trials through the tool of predictive modeling of clinical trial–publication outcomes based on a large data set of clinical trials and associated literature. The adoption of this approach provides a mechanism for both predicting the publication outcome of a given trial and identifying the key factors driving those outcomes.

### Existing Work and Contributions

#### Publication Outcome Studies

Many studies have addressed the publication rates of clinical trials and the factors influencing them. However, previous studies used different statistical analysis methods to examine the association between study characteristics and the publication outcome of a clinical trial. The available studies either analyzed a small number of clinical trials (in the order of hundreds) [3,7,14] or included only clinical trials with specific populations (eg, children or patients with cancer [5,15,16]). Conversely, in our work, we focused on approaching the modeling of publication outcomes *through a predictive lens*, although we also provided a descriptive analysis to better characterize the data set that we developed. Our analysis examined factors that may affect the publication outcome without any constraints regarding the population or medical specialty and, therefore, was more general.

A number of studies have focused on analyzing and remedying the quality of linkage between ClinicalTrials.gov and PubMed [17-22]. The presence of incomplete links may hamper efforts to measure publication and outcome reporting biases and identify relevant trials for systematic reviews. As a result of this, semiautomated methods that rank articles using natural language processing (NLP) techniques and allow humans to scan the top-ranked documents are valuable in supporting the effective identification of clinical trial publications [17,18].

#### Factors Affecting Publication

A variety of factors have been identified as influencing publication outcome, which can be summarized as follows: (1) large clinical trials and those with noncommercial funding are more likely to be published [8,13,23]; (2) industry-funded clinical trials are less likely to appear as publications [7]; (3) the likelihood of publication is associated with the direction and significance of study findings [11,24], although whether to assign this publication bias to rejection by journals or the lack of time and interest by the investigators has been disputed [7]; (4) place of conduct of the research may affect the odds of publication [23]; (5) some fields have higher publication rates, for example, neurology and psychiatry [13] (this may in certain cases be related to the existence of subareas, eg, vascular neurology, with niche journals allowing for easier dissemination [25]); and (6) lack of time and resources by the authors, and even disagreement between coauthors, have been mentioned as potential factors in the literature [26] but are not captured directly in the description of clinical trials and, therefore, are difficult to quantify.

#### Completion Status and Drug Approval Studies

Although we are not aware of any work that analyzes publishability within a predictive framework, several related problems have been treated as classification problems [27-29]. One such task is predicting the completion of a clinical trial. Noncompletion can be seen as similar to nonpublication in terms of undesired consequences. A clinical trial that is not completed typically still involves significant financial resources, so it would make sense to ensure that decision makers are aware of the likelihood of termination or nonpublication in the early stages of a clinical trial, potentially allowing for changes in the study design. Admittedly, having such predictive power would mean that the decision makers are shouldered with the additional responsibility of considering the potential for nonpublication and have the ability to interpret the output of such predictive models. Care would also need to be taken on an ongoing basis to mitigate potential biases in the model and its use [30,31].

Another task related to publication outcome prediction is whether a drug intervention studied in a clinical trial will result in the approval of the drug. Machine learning (ML) over structured data has been explored in this context [32-34], relying on features pertaining to drug and trial characteristics as well as those covering commercial figures relating to indication. Lo et al [33] proposed a large data set consisting of approval outcomes of >6000 drug-indication pairs across almost 16,000 phase-2 trials. Although this represents the largest data collection for applying supervised ML to drug approval, our task was more general (concerning clinical trials without needing to identify drug-indication pairs), allowing us to include an even larger number of clinical trials paired with publication outcomes.

In contrast to descriptive studies on publication status, studies on trial completion and drug approval do include textual inputs from trial descriptions in the modeling, which leads to better sensitivity and specificity than using structured features alone [27,35]. These studies generally use relatively simple methods to represent text. Elkin and Zhu [27] included word-embedding features [36,37] in predicting trial completion but only used static word representations rather than more advanced contextualized word representations derived from pretrained language models [38,39]. In drug approval prediction, features constructed over unstructured input data have been studied by Feijoo et al [35], who focused on predicting drug transitions across clinical trial phases. The authors used simple pattern matching to develop an eligibility criteria complexity metric defined in terms of the number of inclusion and exclusion criteria. Although these criteria were shown to be useful (a higher number of criteria has been connected with a higher risk of trial failure), their representation is still rather rudimentary. In our work, we included the eligibility criteria using state-of-the-art NLP techniques that can capture the meaning of the eligibility criteria.

#### Contributions

We constructed and made available a new data set that provides publication outcomes for trials registered in ClinicalTrials.gov. It is the largest data set of its kind to date.

Predicting the publication status of a clinical trial using numerical, categorical, and textual input features in a single ML model leads to a classification performance of an area under the curve (AUC) of >0.7. We found that textual descriptions of registered trials are an important source of information and are effectively represented using NLP techniques.

We identified a lack of studies investigating publishability within a *predictive* framework. Thus, we confirmed several factors known from *descriptive* studies to influence the publication outcome and identified *new* ones from textual descriptions of clinical trials (eg, eligibility criteria). Our work lays the foundation for a technology that would support trial planning and decision-making by providing, for a given trial, the prominent features that lead to a particular publication outcome. How such technology can best benefit trial developers in increasing the value of their prospective study should be a subject of future research.

## Methods

### Constructing a Data Set Automatically

We used 2 primary resources in our work: the largest available registry of clinical trials, ClinicalTrials.gov, and MEDLINE, a bibliographic database of academic journal articles. For both data sources, we used the data dumps in XML available as of the start of our study in August 2020 [40,41]. To find out which clinical trials were actually published, we adopted a 2-step procedure and took the union over clinical trial-publication links found at each step. The first step recognized all PubMed article IDs directly listed in the registry of clinical trials. However, as some clinical trials lacked this information, we also looked for clinical trial–related information within the publications themselves (second step). We located that information in MEDLINE inside the databank list, from which we retrieved the clinical trial identifier provided that the databank name equaled “clinicaltrials.gov.” To consider a trial published, we required that there be at least one publication associated with it in MEDLINE. If a trial had more than one associated publication, additional pairs were created for each publication.

**Figure 1:**
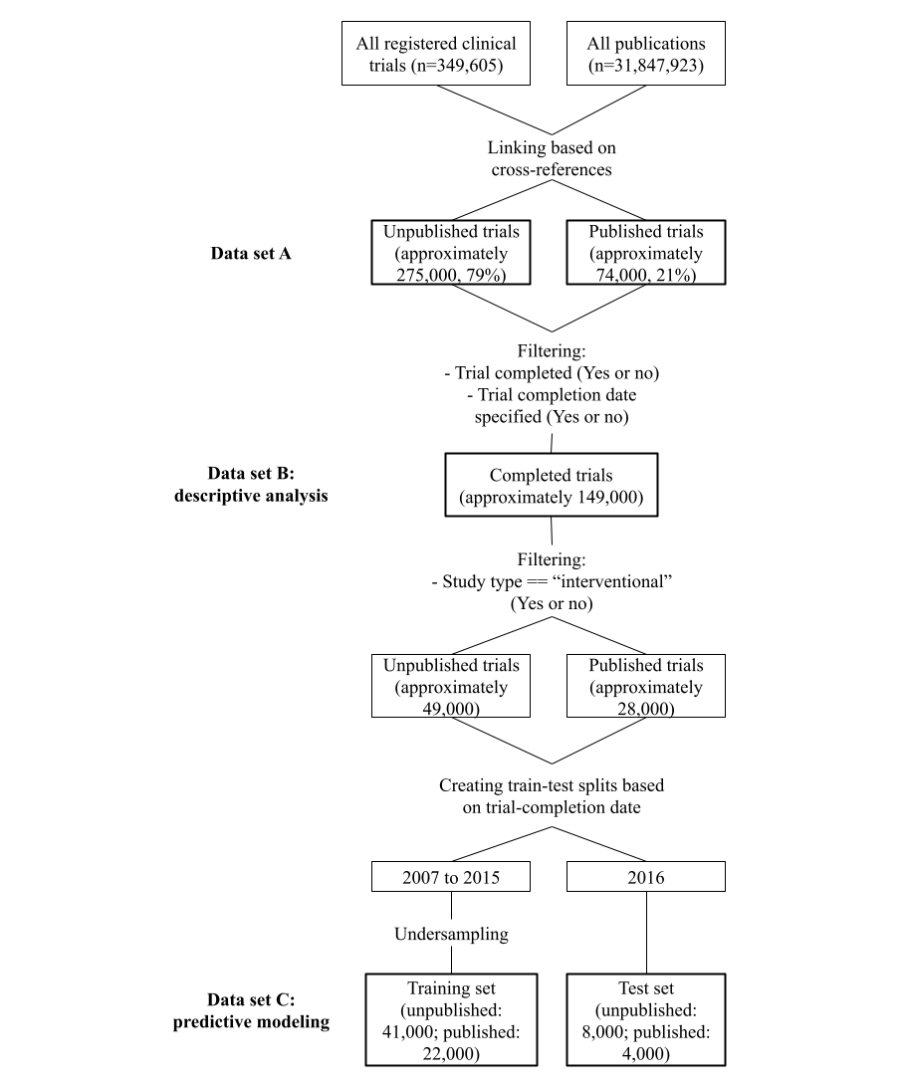
Data set construction.

The final result was a map between clinical trial IDs and PubMed article ID values (*trial-publication map*). In our data set, the number of clinical trials that had an associated publication was 74,394, and there were approximately 275,000 clinical trials without publication, totaling approximately 349,000 trials (data set A). We illustrate the data creation procedure in Figure 1. We made the mapping openly available to promote further work on this topic.

The complete list of data fields and model features used in our work is shown in Table S1 in Multimedia Appendix 1 [42]. Although most of the features were obtained directly from the trial file, information such as the number of research sites and the number of primary or secondary outcomes was not explicitly stated. Therefore, we added those features as they pertain to clinical trial design and may contain an important signal for the prediction of publication status.

The data set used in our descriptive analysis and predictive modeling (*data set B*) was based on selecting the instances that satisfied a few additional criteria. Specifically, we filtered out data instances that did not satisfy the following two conditions: (1) the study had both started and been *completed*, with known start and end dates and without “anticipated” status (as the information about a clinical trial may be updated several times after registration, such as updating the enrollment field, which indicates the planned number of participants, the information remains stable after completion, thus increasing the representativeness); and (2) the *completion date* of the study was later than 2006 (to remove older studies whose information was less complete) but earlier than 3 years before our data collection (to allow time for publication, similarly to Jones et al [7] and Ross et al [3]).

Performing these steps reduced the size of the data considerably. The resulting data set was used to obtain the descriptive statistics.

In addition, we constrained the type of study to be *interventional* to obtain the data set used in predictive modeling (*data set C*). We decided to exclude observational studies as they are less common and are characterized by several features that are different from those of interventional studies.

To emulate the real-world scenario of predicting publishability of future trials, we partitioned the data such that the completion dates of all trials in the test set postdated those in the training data set. This also made the task more challenging as we could expect previously unseen interventions in the test set. Finally, we removed all features from each trial record that would not have been known at the time of registration of the trial, such as the trial duration and results. Although including them would simplify the prediction, it would also make the task less realistic. By comparison, we note that, in the related ML task of the drug approval prediction work by Lo et al [33], the authors assumed that the same information about clinical trials *is* accessible. As these features are found to be strong predictors of drug approval, the predictive performance is likely to suffer in the more realistic scenario of this information not being available.

As the number of unpublished clinical trials in data set C was much larger than that of published clinical trials, we randomly undersampled the unpublished trials for our publication prediction experiments. We performed the undersampling by stratifying per completion year, keeping roughly equal percentages of positive and negative labels in each year. Note that we performed this step for the training set only, preserving the real-world label bias in the test set, again to make the task as faithful to reality as possible.

### Manually Constructed Test Set

The aforementioned data construction approach provided a large-scale data set that allowed us to analyze and predict the publication status at scale using ML models. However, some links between clinical trials and publications may be incomplete, as we mentioned in the *Existing Work and Contributions* section. Therefore, we gathered data from 3 previously published studies [3,18,20] that included manual publication status annotations (see Table 1 for the statistics). Although the scale of these annotations was smaller than in our automatically constructed data set, because of human effort, it was less likely that the publication of a clinical trial would go unnoticed. We used this data set as an additional test set and also made it publicly available with the permission of the original authors [43].

**Table 1:** Data from previously published studies. A total of 5 studies were included in more than one original work but received the same annotation. Owing to this, the size of the resulting test set was less than the sum of the sizes of the individual data sets.

	Size	Proportion of positive labels (“published”) out of all
Ross et al [3]	630	0.54
Zarin et al [20]	148	0.23
Dunn et al [18]	199	0.45
Combined	972	0.48

### Modeling Approach

To study factors associated with publication status and learn to predict whether a clinical trial is likely going to be published, we created 3 types of features for our models: numerical, categorical (both can be seen as structured inputs), and textual features. The textual features encode a wealth of information that augments the structured information and have the potential to improve predictive modeling, but they are also potentially much noisier. An example of textual fields that can be indicative of publication status are the inclusion and exclusion criteria. A possible link between eligibility criteria, sample size, significant effect, and publication status has been pointed out by Elkin and Zhu [27]. NLP techniques allowed us to extract and represent this information in a predictive model as well as highlight which textual features are important.

As a simple baseline, we used a k-nearest neighbor classifier that only used numerical and categorical features (with no text-based features). At test time, the classifier predicts the predominant label among *k* training instances that are closest to the test instance in terms of Euclidean distance. Through a random search over various values of *k*, we settled on *k*=460.

We trained and evaluated 2 different models that incorporated textual features: a random forest (RF) classifier and a neural network (NN).

For RF, a standard approach to include textual inputs is to convert them into numeric word vectors, extracting both unigrams and bigrams. These terms are weighted using term frequency-inverse document frequency (Schütze et al [44]), whereby the frequency of a term in a document is divided by the proportion of documents that that term appears in within the data set to down-weight common terms. We thresholded the vocabulary by selecting the 20,000 most frequent terms. We used the one-hot encoding method to represent categorical features and included numeric features without additional adaptation. We report other RF details in Multimedia Appendix 2.

In the NN, the categorical features are embedded using a weight matrix that is randomly initialized and updated during training. The textual inputs (examples are included in Table 2) are embedded using pretrained language models that output context-dependent token activations [39], as explained in more detail next.

**Table 2:** Examples of selected textual features from clinical trial metadata.

Feature name and identifier			Textual excerpt
**Brief title**			
	NCT01309919	Bleeding Patterns and Complications After Postpartum IUD Placement: a Pilot Study	
	NCT00230971	Study Comparing Tigecycline Versus Ceftriaxone Sodium Plus Metronidazole in Complicated Intra-abdominal Infection (cIAI)	
	NCT01364948	Effect of Coconut Oil Application in Reducing Water Loss From Skin of Premature Babies in First Week of Life (TEWL) (TopOilTewl)	
**Brief summary**			
	NCT01309919	The purpose of the study is to determine the feasibility of placing the levonorgestrel-releasing intrauterine system (LNG - IUS, Mirena®) post-delivery. The investigators will gain information about complications at the time of placement; the investigators will also examine the expulsion rate, side effects, bleeding patterns and subject satisfaction at various time periods after insertion.	
	NCT00230971	This is a study of the safety and efficacy of tigecycline to ceftriaxone sodium plus metronidazole in hospitalised subjects with cIAI. Subjects will be followed for efficacy through the test-of-cure assessment. Safety evaluations will occur through the treatment and post-treatment periods and continue through resolution or stability of the adverse event(s).	
	NCT01364948	The skin of newborn infants is immature and ineffective as a barrier. Preterm skin exhibits even more vulnerability to the environment due to poor self regulatory heat mechanisms, paucity of fatty tissue and its thinness. Most preterm babies lose up to 13\% of their weight as water loss from their skin during the first week of life. Many strategies have been utilised by neonatologists to decrease this water loss. Oil application on the skin can act as a non permeable barrier and can help in reducing water loss from the skin. Edible coconut oil, often used for traditional massage of babies by Indian communities, is culturally acceptable and Hence the investigators decided to undertake this study to objectively assess the reduction in water loss from skin after oil application	
**Inclusion criteria**			
	NCT01309919	Age 18 years or older, speak either English or Spanish, desire to use an IUD as their postpartum contraception (IUD arm), do NOT desire an IUD as their contraception (Diary Only arm), plan to deliver at Baystate Medical Center	
	NCT00230971	Clinical diagnosis of complicated intra-abdominal infection that requires surgery within 24 hours. Fever plus other symptoms such as nausea, vomiting, abdominal pain.\\	
	NCT01364948	All preterm babies born at the study center with birth weight 1500gms were eligible for inclusion in the study.	
**Participant condition**			
	NCT01309919	Postpartum period	
	NCT00230971	Appendicitis, cholecystitis, diverticulitis, intra-abdominal abscess, intra-abdominal infection, and peritonitis	
	NCT01364948	Trans Epidermal Water Loss (TEWL)	
**Keywords**			
	NCT01309919	Intrauterine device, Mirena, levonorgestrel intrauterine system, postpartum contraception	
	NCT00230971	Intra-abdominal infections, abscess	
	NCT01364948	Preterm, VLBW, coconut oil application, transepidermal water loss, weight gain	

We evaluated the RF and NN classifiers that used textual features compared with those without, in which only structured features were used.

We opted for 2 different encoders: Bidirectional Encoder Representations from Transformers (BERT) [39], pretrained on general-domain English corpora, and BERT for scientific texts (SciBERT) [38], pretrained on the biomedical domain. We used the same idea as Adhikari et al [45], who took the hidden layer output at the sentence-level classification level as the representation of the document. In addition, we used the hidden outputs of the 3 last layers [46] as inputs to the top dense layers of our classifier. To refine the model’s representational capacity, we included 2 additional sources of information: positional and segmental. For the first one, a trainable positional embedding [47], which is unique to each token, is added to the token vector to endow the model with a sense of word order. For the second one, a trainable segment embedding helps the encoder discriminate between the multiple, independent textual fields (Table S1 in Multimedia Appendix 1) that are passed to the model as one long string of text. We found the interchangeable segment scheme illustrated in Figure S1 in Multimedia Appendix 1 to work best. Another variation represents each text field with a different segment embedding but works less well, although the difference is small. In addition, an alternative scheme for positional embeddings in which the embedding index is restarted with each text field yields similar results. We took inspiration for that from Herzig et al [48], who used positional embeddings in the context of table parsing to enhance input structuring.

A limitation of the original BERT architecture is that it can only accept sequences of up to 512 tokens. Therefore, we needed to truncate the textual inputs exceeding this limit. We started by selecting the first n=512/*T* tokens of each field (*T* being the total number of textual fields to encode). As some textual fields can be shorter, we progressively raised *n* across all fields until we reached the maximum number of tokens. Finally, the parameters of the encoder were fine-tuned jointly with the remaining NN parameters on our publication outcome prediction data set, minimizing the cross-entropy loss during training.

In addition to adopting the standard BERT model in the NN, we looked at 2 adaptations of the training regime: a special case when the encoder parameters are left unchanged during training (named “frozen” in the table of results) and a model that receives cased text as input (“cased”; ie, text that has not been previously lowercased), the latter being the most common practice. Finally, for RF, we tested an adaptation that, instead of the term frequency-inverse document frequency encoder, uses language model representations previously induced in the text. These representations were kept fixed throughout the training and testing phases.

### Evaluation Details

We evaluated the predictive performance using the *F*_1_-score measure (*F*_1_ = 2 × [P × R / (P + R)]), which is the harmonic mean of precision (P = TP / [TP + FP]; the proportion of trials predicted as published out of all predictions, where TP are true positives and FP are false positives) and recall (R = TP / [TP + FN]; the proportion of trials predicted as published out of all published trials, where FN are false negatives). We also reported the area under the receiver operating characteristic curve (itself indicative of the trade-off between recall and false-positive rate at various thresholds over the predicted probabilities), which was useful in summarizing the classifier’s ability to distinguish between classes via a single figure of merit.

## Results

### Descriptive Analysis

#### Overview

To obtain a clear idea of the *publication rate* in our data set, we plotted the number of published and unpublished studies per year, as shown in Multimedia Appendix 3. We observed that the number of registered trials was monotonically increasing (with >20,000 trials registered in 2016), but the number of published trials increased less strongly. For trials with an earlier completion year, the publication rate was approximately 45%, whereas, for later trials, it decreased by approximately 10%. For comparison, existing studies on publication rates reported highly variable publication percentages, up to 77% in Huiskens et al [6] and as low as 11% in Chen et al [9] depending on the medical area and length of follow-up considered.

**Figure 2:**
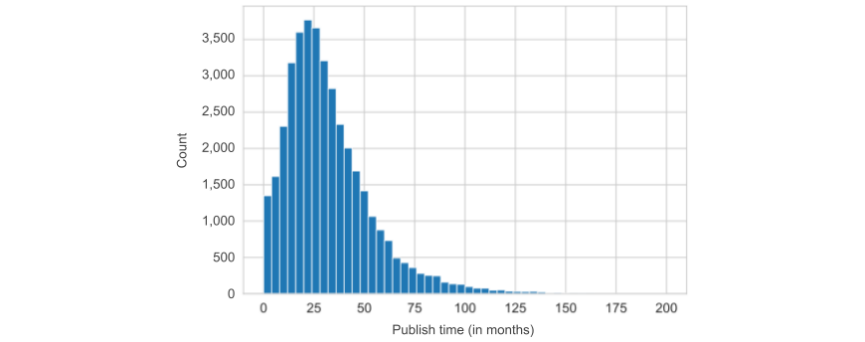
The distribution of publication times in months.

Furthermore, we examined the *time needed to publish*. Analyzing only the published studies, we found a median time to publish of 27 months. We show the distribution of publication times in Figure 2. For a smaller number of trials, it can take much longer to publish, as seen by the long tail on the right of the plot. The previous studies generally reported shorter times of approximately 19 to 23 months [3,9,16].

**Figure 3:**
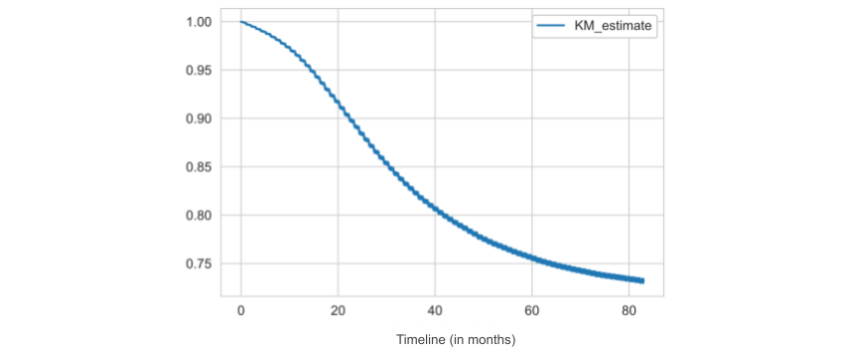
A Kaplan-Meier (KM) plot representing the probability (y-axis) that a trial will go unpublished for longer than the number of months shown on the x-axis.

An additional way of analyzing publication time is to plot the probability that a study will go unpublished for an interval longer than some time *t*. We borrowed here a tool from survival analysis, the Kaplan-Meier plot. By analogy, the survival time in our case represents the time that a clinical trial remains unpublished, and the relevant event is the publication. Some individuals (clinical trials) may be lost to follow-up (right censoring), which is also considered by the method. We see in Figure 3 that, when given a very short period (eg, a few months after completion), the chance is still high that the trial will not be published. When given more time, the probability of nonpublication drops, although it remains fairly high even for very long intervals (at 80 months, it is still >70%).

#### Association Between Publication Outcome and Categorical Features

To analyze the relationship between a feature and the publication outcome, we applied the chi-square test (in line with the related literature [8,9,14,16,23,49,50]) but, because of its sensitivity to the sample size [51,52], we also carried out the Cramér *V* association test for discrete variables. In this analysis, we followed the related work and focused on categorical features only. In the *Predictive Performance* section, we analyze the importance of all feature types in predictive performance. The results for all categorical features are shown in Table 3. The features with the highest values of *V* include the overall status (eg, a value such as “Suspended” may be indicative of future publication), whether the results were reported, enrollment type (anticipated vs actual), and the phase of the trial (when calculating the odds ratio over different phases of the trial, we found that trials in phase 3 were 2 times more likely to be published than trials in other phases). By contrast, some features such as the type of observational study (retrospective, prospective, or cross-sectional) and the class of funding agency (US National Institutes of Health, other US Federal agencies, industry, or other) can hardly be associated with publication status. The latter example is particularly surprising as most previous works have reported that the source of funding is a strong indicator of publication status [8,23,50], with the exception of Gandhi et al [14].

**Table 3:** Strength of association between categorical features extracted directly from structured metadata associated with clinical trials and publication status. For the definition of each feature, see Table S1 in Multimedia Appendix 1.

Feature name	Chi-square *P* value	Cramér V
overall_status	.001	0.26
were_results_reported	.001	0.157
enrollment_type	.001	0.153
Phase	.001	0.126
plan_to_share_ipd	.001	0.095
intervention_type_behavioral	.001	0.06
has_dmc	.001	0.056
intervention_model	.001	0.053
intervention_type_diagnostic_test	.001	0.047
has_single_facility	.001	0.044
intervention_type_device	.001	0.039
Country	.001	0.035
study_type	.001	0.034
Allocation	.001	0.026
primary_purpose	.001	0.025
is_fda_regulated_device	.001	0.023
Masking	.001	0.022
intervention_type_dietary_supplement	.001	0.021
intervention_type_biological	.001	0.019
Gender	.001	0.018
intervention_type_combination_product	.001	0.017
intervention_type_other	.001	0.016
intervention_type_radiation	.001	0.013
sampling_method	.001	0.013
intervention_type_drug	.001	0.012
intervention_type_procedure	.001	0.012
observational_model	.002	0.012
is_us_export	.13	0.011
responsible_party_type	.001	0.011
intervention_type_genetic	.001	0.01
healthy_volunteers	.001	0.009
is_fda_regulated_drug	.001	0.009
observational_prospective	.14	0.006
agency_class	.32	0.002

### Predictive Performance

#### Overview

**Figure 4:**
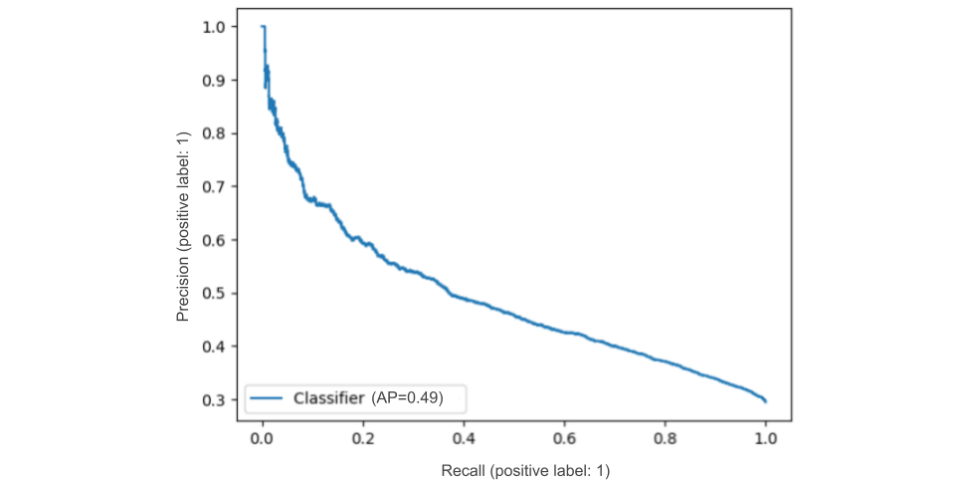
Precision-recall curve for the positive class (publication) using the neural network model with structured and textual features from a Bidirectional Encoder Representations from Transformers model for scientific texts. AP: average precision.

**Figure 5:**
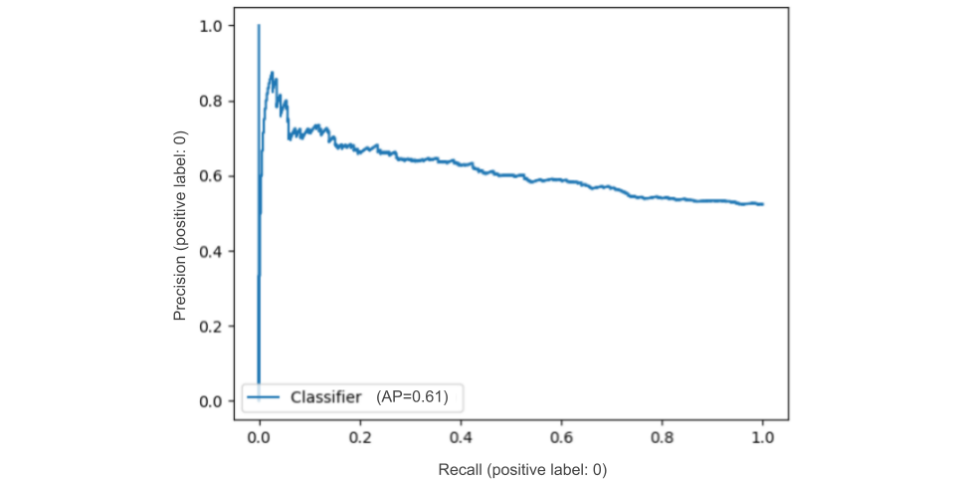
Precision-recall curve for the negative class (nonpublication) using the neural network model with structured and textual features from a Bidirectional Encoder Representations from Transformers model for scientific texts. AP: average precision.

The main results of our predictive models for data set C are shown in Table 4. Interestingly, the k-nearest neighbor baseline already set a high bar for the use of structured inputs. We see that the best performance on the test set was achieved with the models that used textual information. The 2 evaluation metrics show slightly different trends (ie, when looking at *F*_1_-score, the neural models using BERT-based representations performed better than the RF classifier using the bag-of-words representation); however, according to AUC, the RF classifier outperformed different variants of the neural model. Judging by the improvement obtained when including the textual features in both models, the NN model makes more effective use of these features. We found that the difference between the NN model using only structured features and the NN model using SciBERT-encoded text features was statistically significant at *P*<.001 (statistic value: 778.4), measured with the McNemar test for binary classification tasks [53]. Although it had a considerably lower performance compared with the RF classifier when including only the structured features, the performance difference between the 2 models vanished when including the textual features. For the neural model, choosing a BERT model with a better domain fit (ie, SciBERT) appears to boost *F*_1_-score, but the differences are too small to make a judgment in the case of AUC. We include the precision-recall curves in Figures 4 and 5, calculated using the predictions of the model that tested best in terms of *F*_1_-score (ie, NN with structured and SciBERT textual features).

**Table 4:** Results for publication prediction^a^.

Method	Input	Validation		Test	
		*F*_1_-score	AUC^b^	*F*_1_-score	AUC
K-nearest neighbor	Structured	0.592	N/A^c^	0.611	N/A
RF^d^	Structured	0.64	0.701	0.614	0.704
RF	Structured+text (TF-IDF^e^)	0.656	0.721	0.623	0.719
RF	Structured+text (SciBERT^f^)	0.65	0.709	0.63	0.711
NN^g^	Structured	0.611	0.672	0.607	0.612
NN	Structured+text (frozen SciBERT)	0.642	0.689	0.63	0.696
NN	Structured+text (SciBERT)	0.648	0.708	0.641	0.7
NN	Structured+text (cased SciBERT)	0.641	0.697	0.637	0.701
NN	Structured+text (BERT^h^)	0.64	0.699	0.633	0.7

^a^All models use categorical and numerical features (“structured”). When textual features are added, this is marked with “+ text.” As the k-nearest neighbor classifier does not output probabilities, we cannot calculate the area under the curve.

^b^AUC: area under the curve.

^c^N/A: not applicable.

^d^RF: random forest.

^e^TF-IDF: term frequency-inverse document frequency.

^f^SciBERT: Bidirectional Encoder Representations from Transformers model for scientific texts.

^g^NN: neural network.

^h^BERT: Bidirectional Encoder Representations from Transformers.

#### Factors Affecting Publication

To determine which features play a key role in prediction, we used a feature permutation technique to obtain the features ranked by their respective drop in performance. We performed this analysis using RF only because of faster inference times. The classifier is trained once; then, at test time, a corrupted representation of a feature is obtained by shuffling its possible feature values in the test set. After that, the model is applied to the test set, and the drop in accuracy is calculated compared with the performance on the noncorrupted data set. We only corrupted one feature at a time and repeated the process for all features. The entire process was performed 5 times using different random seeds for shuffling, after which the reported scores were averaged.

The results, organized according to feature type, are shown in Table 5. The most significant numerical feature is the number of enrolled participants, with a possible explanation being that it may affect the reliability of the results (thus ultimately increasing the odds of publication). Similarly, a larger number of facilities has been linked to higher publication rates [8]. The number of outcomes indicates the size and complexity of the study, which may in turn also affect publishability. For textual inputs, the narrative describing the trial (the detailed description and brief summary) as well as the eligibility criteria are the strongest features. We observed that some textual features contained overlapping information. For example, the brief title could be subsumed into the official title. The same word often occurred in different inputs, and this redundancy can be a strong indicator for predicting publication status. For example, when we measured the importance of the words in RF using the impurity criterion of our RF implementation [9], we found that the presence of *randomized* (occurring in both the official title and detailed description) was a strong discriminator between published and unpublished studies.

In the case of categorical inputs, we found similar features to be important, as mentioned in the *Descriptive Analysis* section, including the country of the main institution (“country”) and whether the study had a data monitoring committee (“has dmc”). However, some features that were found to be important in our descriptive analysis and in the prior work were less important in the predictive approach (eg, the phase of investigation [“phase”], the allocation of participants to trial arms [“allocation”], and the method used to assign an intervention to participants [“intervention model”]).

**Table 5:** The drop in accuracy after permuting the values of a feature as measured with random forest using term frequency-inverse document frequency representation of text. The values for each feature type are ranked in decreasing order, so the most important features are mentioned first.

Feature type and feature			Drop in accuracy
**Numerical**			
	number_of_facilities	0.007364	
	outcome_counts_secondary	0.004911	
	outcome_counts_others	0.004068	
	outcome_counts_primary	0.003702	
	number_study_directors	0.003518	
	number_study_chairs	0.003359	
	minimum_age	0.003235	
	number_principal_investigators	0.003157	
	maximum_age	0.002719	
	number_of_arms	0.000985	
**Textual**			
	detailed_description	0.010193	
	brief_summary	0.008551	
	criteria_Exclusion	0.008313	
	criteria_Inclusion	0.004971	
	official_title	0.003428	
	brief_title	0.001433	
	Source	0.001342	
	responsible_party_keywords	0.001064	
	participant_condition	0.00064	
**Categorical**			
	has_single_facility	0.004591	
	intervention_type_Behavioral	0.004211	
	primary_purpose	0.003914	
	Country	0.003804	
	intervention_type_Biological	0.003643	
	is_fda_regulated_device	0.003376	
	is_us_export	0.003333	
	intervention_type_Diagnostic_Test	0.003322	
	intervention_type_Combination_Product	0.003322	
	intervention_type_Genetic	0.003322	
	is_fda_regulated_drug	0.003321	
	intervention_type_Procedure	0.003205	
	has_dmc	0.003185	
	intervention_type_Other	0.003144	
	intervention_type_Radiation	0.003144	
	intervention_type_Device	0.003078	
	Gender	0.003012	
	responsible_party_type	0.002925	
	intervention_type_Dietary_Supplement	0.002873	
	plan_to_share_ipd	0.002819	
	healthy_volunteers	0.002607	
	intervention_type_Drug	0.00227	
	agency_class	0.001854	
	Phase	0.001426	
	Allocation	0.001347	
	intervention_model	0.00131	

#### Performance on the Manually Verified Test Set

As an additional experiment, we took the model that achieved the highest *F*_1_-score on the automatically constructed data set (NN with structured+text [SciBERT] input features) and applied it to the test set built from the manually verified publication links introduced in the *Manually Constructed Test Set* section. We measured an *F*_1_-score of 55.9 and area under the receiver operating characteristic curve of 58.6. To better understand this drop in performance with respect to automatically obtained test sets, we calculated a confusion matrix, which revealed that the model too eagerly predicted “publication” (ie, it was more likely to commit a type-1 error [a false positive, 272/972, 28% of the time] than a type-2 error [a false negative, 146/972, 15% of the time]). As the test data consisted of 3 subsets, there might be important individual variations in the performance that we need to consider. Indeed, splitting the results according to each subset (Table 6), we noticed that the subset from Zarin et al [20] showed lower performance than the subsets from Ross et al [3] and Dunn et al [18], both with similar performance. Our explanation is that these subsets contain varying proportions of positive labels, which, if different from those seen during training, will negatively affect the test performance. Specifically, the Zarin et al [20] subset has only 23% (34/148) of positive labels compared with approximately 50% (410/824, 49.8%) in the remaining subsets. Understandably, the model that was trained on roughly equal portions of positive and negative instances overpredicted the positive class on the Zarin et al [20] subset, and almost all modeling mistakes in this case were due to false positives (78/87, 90% compared with 9/87, 10% of false negatives). We found that this negative effect vanished when the model was retrained with a similar ratio of positive to negative instances. We used the nonbalanced version of our training data set (data set C in Figure 1).

**Table 6:** Data statistics and performance on the subsets of the manually verified test set.

	Ross et al [3]	Zarin et al [20]	Zarin et al [20] with nonbalanced training set	Dunn et al [18]
Percentage positive^a^	54	23	23	45
*F*_1_-score	58.4	43.4	58.2	55.0
AUROC^b^	62.3	52.6	53.5	60.4

^a^*Percentage positive* represents the percentage of instances bearing the positive label (*published*) out of all instances.

^b^AUROC: area under the receiver operating characteristic curve.

## Discussion

### Limitations

Although our work established at scale the various attributes associated with a higher publication rate and the positive impact of including textual descriptions of clinical trials in a predictive framework, a few additional considerations are necessary.

The qualitative performance of an ML model is sensitive to the quality of the underlying data that are used for training and testing, and predicting publication success is no different. When constructing our data set, we noticed that incorrect information existed in the trial registration entries (eg, the estimated completion year may be set to 2099). In addition, the current status of the study (eg, ongoing, completed, or terminated) may not be always up to date, and this is similar for other registered information. Incompleteness and incorrect information in ClinicalTrials.gov have been examined in the literature [7,54-56], but the precise extent of this is unknown and difficult to estimate, and it would require substantial manual effort to reveal it. We see noise as an integral part of learning from large data collections, similar to the related work (*Existing Work and Contributions* section) that uses structured resources such as ClinicalTrials.gov [27-29,32-34] and to the work on learning under distant supervision [57-59]. As our classifiers used a very large number of training instances and each instance is represented using multiple features, the effect of occasional noise is deemed small.

Another potential source of noise in our automatically constructed data set could stem from the linkage between clinical trials and their publications, which is established automatically and, hence, prone to incorrect or missed links. The data set was also limited to studies that were publicly available and indexed in public resources. Although conference abstracts and other gray literature resources may provide additional context on trial outcomes, they are not typically considered to be formal publications and require ad hoc strategies for collection that are beyond the scope of our study. Overall, the results presented reflect the most realistic scenario possible based on accessible resources.

Finally, a more general limitation in the modeling of publication outcomes is that it is difficult to capture and quantify the influence of factors that are not available in trial registries but would otherwise be useful, particularly for understanding nonpublication, for example, whether investigators did not have enough time to publish and instead focused on other tasks, whether there were changing interests or disagreements between coauthors, whether researchers believed that a journal was unlikely to accept their work, and whether financial problems or other contractual issues prevented publication [15,60-62]. Although such information is obtainable from study authors in principle, it would be extremely difficult to carry out such information acquisition at scale, and it is not currently available in public resources.

### Impact

In this study, we sought to simulate a real-world situation in which a prospective estimate is desired regarding the publication outcome of a clinical trial. To this end, we carried out a set of experiments on the newly created data set that linked clinical trial records from the period of 2007 to 2016 with their publications, if they existed, with a follow-up period of 4 years. The resulting data set represents the largest such collection available to date. We have shown how a combination of heterogeneous features—including text features derived from the clinical trial registry record—can lead to a classification performance of >0.7 AUC; this means that, if one randomly selects a case that is positive (ie, a trial that will eventually lead to publication), there is at least a 70% chance that the case is also classified as such. This technology has strong potential to be used in trial design. It can provide a prospective estimate of publishability in the early stages of a clinical trial when the properties of the study design and environment are already known, more broadly giving an indication of the viability of the trial. The tool could reveal to trial developers the different areas suggestive of lowered publication chances (and, by extension, of a reduced value of their study) before wasting resources unnecessarily. In future work, we will explore the incorporation of this model into a system that can effortlessly and in a human-friendly way provide, for a given trial, the prominent features that lead to a particular outcome, as well as indicate the reliability of the classifier’s decision, to support trial planning and decision-making.

## Appendix

Multimedia Appendix 1 Text-representation scheme.

Multimedia Appendix 2 Experimental details.

Multimedia Appendix 3 The distribution of published and unpublished trials per year of completion.

## Abbreviations

AUC: area under the curve
BERT: Bidirectional Encoder Representations from Transformers
ML: machine learning
NLP: natural language processing
NN: neural network
RF: random forest
SciBERT: Bidirectional Encoder Representations from Transformers model for scientific texts

## Data Availability

We have made the data set publicly available [63].
