# Supplementary figures and images for: Predicting Publication of Clinical Trials Using Structured and Unstructured Data: Model Development and Validation Study
Source: J Med Internet Res. 2022 Dec 23;24(12):e38859. doi: 10.2196/38859 (PMC9823568; doi:10.2196/38859)

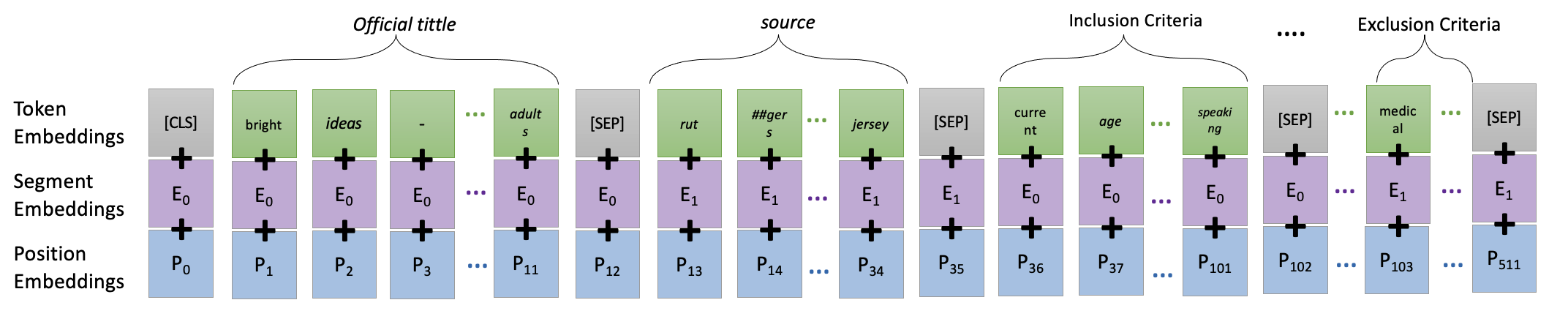

Supplement: Multimedia Appendix 1 [file jmir_v24i12e38859_app1.png]

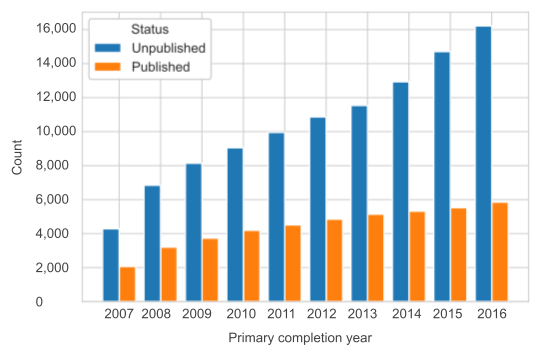

Supplement: Multimedia Appendix 3 [file jmir_v24i12e38859_app3.png]
