# Supplementary material for: Predicting Publication of Clinical Trials Using Structured and Unstructured Data: Model Development and Validation Study
Source: J Med Internet Res. 2022 Dec 23;24(12):e38859. doi: 10.2196/38859 (PMC9823568; doi:10.2196/38859)
Supplement: Multimedia Appendix 2 [file jmir_v24i12e38859_app2.docx]

## Appendix

### A.1 Experimental details

#### Neural end-to-end model

In the implementation of our models, we have used the PyTorch library and the pretrained models at http://huggingface.co. We trained our models using four Nvidia V100 GPUs with the distributed data-parallel method (PyTorch-Lightning [42]).

Based on manual examination of results in the validation set, we determined the following hyper-parameters for our neural end-to-end model. We set the batch size to 24 (16, 24, 36) and use the Adam optimiser with a learning rate of 2e-5 (5e-5, 3e-5, 2e-5), and the epsilon parameter for numerical stability to 1e-8. We disabled the warm-up phase in the learning rate scheduling.

#### Bag-of-words classifier

We use the scikit-learn implementation of the random forest model. We perform random search to choose the hyper-parameters, exploring the following options: n estimators (10–1000), max features (“sqrt”, “log2”), max depth (10–110), min samples split (2–20), min samples leaf (2–20), bootstrap (True, False).

*Table A1: List of features and their description.*

| Numerical | number_study_chairs | number of study chairs |
| --- | --- | --- |
|  | number_principal_investigators | number of principal investigators |
|  | number_study_directors | number of study directors |
|  | outcome_counts_primary | number of primary outcomes |
|  | outcome_counts_secondary | number of secondary outcomes |
|  | outcome_counts_others | number of other outcomes |
|  | minimum_age | minimum age of participants |
|  | number_of_facilities | number of organisations where the clinical study is being conducted |
|  | maximum_age | maximum age of a participant to be eligible for the clinical study |
|  | enrollment | number of estimated or actual participants |
|  | number_of_arms | number of arms |
|  | number_of_groups | number of groups |
| Categorical | phase | numerical phase of a clinical trial involving a drug product |
|  | agency_class | type of funding agency |
|  | country | country of the main institution |
|  | study_type | nature of the investigation (interventional, observational, expanded access) |
|  | allocation | method by which participants are assigned to arms in a clinical trial |
|  | plan_to_share_ipd | whether there is a plan to share individual participant data (IPD) |
|  | primary_purpose | main aim of the intervention(s) being evaluated by the clinical trial |
|  | has_dmc | whether a study has a data monitoring committee (DMC) to periodically review the collected data and give advise on whether the trial should be modified |
|  | enrollment_type | whether the enrollment number is actual or anticipated |
|  | gender | sex of the participant eligible for the clinical trial |
|  | healthy_volunteers | whether healthy volunteers may participate in the study |
|  | responsible_party_type | whether the responsible party is the sponsor, PI or sponsor-investigator |
|  | is_fda_regulated_drug | whether the clinical trial is studying an FDA-regulated drug product |
|  | is_fda_regulated_device | whether the clinical trial is studying a device product |
|  | intervention_model | method to assign interventions to participants |
|  | sampling_method | method used for the sampling approach in observational studies |
|  | is_us_export | whether the drugs or devices in the clinical trials are produced by US and are exported to another country |
|  | has_single_facility | whether only one facility is responsible for the clinical trial |
|  | observational_model | main method to identify participants and follow-up |
|  | observational_prospective | observational type of observational studies |
|  | intervention_type_device | whether the general type of intervention concerns a device |
|  | intervention_type_biological | whether the general type of intervention is biological |
|  | intervention_type_dietary | whether the general type of intervention includes dietary supplements |
|  | intervention_type_behavioral | whether the general type of intervention is behavioural |
|  | intervention_type_combination | whether the general type of intervention is a combination product |
|  | intervention_type_diagnostic | whether the general type of intervention includes a diagnostic test |
|  | intervention_type_genetic | whether the general type of intervention is genetic |
|  | intervention_type_drug | whether the general type of intervention concerns drugs |
|  | intervention_type_procedure | whether the general type of intervention concerns a procedure or surgery |
|  | intervention_type_radiation | whether the general type of intervention concerns radiation |
|  | intervention_type_other | whether the general type of intervention is other than those listed above |
| Textual | brief_title | short title (limited to 300 characters) of the clinical trial |
|  | official_title | title in the protocol |
|  | brief_summary | brief description of the clinical trial |
|  | detailed_description | long description of the clinical trial |
|  | participant_condition | condition or primary disease of participants |
|  | source | name of the responsible party |
|  | responsible_party_keywords | words for describing the protocol |
|  | criteria_inclusion | list of criteria for selecting participants for the study |
|  | criteria_exclusion | list of criteria for selecting participants for the study |
